# Wearable technologies for perioperative recovery monitoring in lung cancer surgery: a systematic review of feasibility, recovery outcomes, and evidence certainty

**DOI:** 10.3389/fonc.2026.1832349

**Published:** 2026-06-03

**Authors:** Xiang Lin, Jingwen Zhang, Beinuo Wang, Zhenghao Dong, Yu Tong, Jian Zhou, Hu Liao

**Affiliations:** 1 Department of Thoracic Surgery, West China Hospital, Sichuan University, Chengdu, China; 2 West China School of Medicine, Sichuan University, Chengdu, Sichuan, China

**Keywords:** digital biomarkers, ERAS, lung cancer surgery, perioperative monitoring, prehabilitation, wearable devices

## Abstract

**Background:**

Enhanced Recovery After Surgery (ERAS) pathways in thoracic oncology emphasize early mobilization and objective discharge readiness, but perioperative functional recovery is often assessed intermittently. Wearable devices may provide continuous, objective recovery metrics.

**Methods:**

We conducted a PRISMA 2020-based systematic review registered in PROSPERO (CRD420261325339). PubMed, Scopus, and Web of Science Core Collection were searched for English-language studies published between February 2, 1996 and February 2, 2026. Eligible reports included adults undergoing lung cancer surgery or clinically relevant pulmonary resection and evaluated wearable-based activity, physiologic monitoring, or rehabilitation support across the preoperative, in-hospital, or post-discharge phases. Risk of bias was assessed using RoB 2, ROBINS-I, or design-appropriate feasibility and measurement appraisal. Certainty of evidence was qualitatively informed by GRADE principles, and findings were synthesized narratively because of clinical and methodological heterogeneity.

**Results:**

Eight reports representing seven independent cohorts were included: two randomized trials, one nonrandomized trial with historical controls, two prospective observational studies, two companion single-arm preoperative feasibility/effectiveness reports, and one development/usability agreement study. In the Move For Surgery RCT, wearable-enhanced preconditioning reduced prolonged hospital stay >5 days from 24% to 7% (12/50 vs 3/45; *p*=0.021). A digital chest drainage RCT reported shorter postoperative length of stay and chest tube duration in the intervention group, although the cohort was not restricted to lung cancer. Observational studies showed weak but significant associations between perioperative step counts and recovery outcomes. Feasibility studies supported device use and data transmission, while a smartwatch-ePRO study showed close agreement with electronic health record measurements.

**Conclusions:**

Wearable-based perioperative monitoring appears feasible and may provide objective recovery signals in lung cancer surgery. However, current evidence remains sparse, heterogeneous, and often indirect. Findings should be interpreted as hypothesis-generating rather than sufficient to support routine clinical implementation.

**Systematic Review Registration:**

https://www.crd.york.ac.uk/PROSPERO/view/CRD420261325339, identifier CRD420261325339.

## 1 Introduction

Enhanced recovery after lung surgery is driven by multiple evidence-based measures, including early mobilization, opioid-sparing analgesia, minimization of tubes, and optimized discharge readiness. Enhanced Recovery After Surgery (ERAS) is defined as a multimodal perioperative care framework incorporating patient education, early mobilization, opioid-sparing analgesia, respiratory physiotherapy, optimized chest tube management, early oral intake, and objective discharge readiness. However, many of these targets are measured intermittently, often relying on subjective reports or coarse ward documentation (1). This creates a perioperative “measurement gap,” particularly for functional recovery and symptom-driven volatility in the early postoperative period and after discharge.

Wearable devices were defined as patient-worn, body-attached devices capable of passively or semi-passively collecting objective activity or physiologic data. Eligible devices included pedometer-like activity trackers, consumer fitness trackers, research-grade accelerometers, smartwatches, and multimodal wearable systems paired with electronic patient-reported outcomes (ePROs). These metrics may serve three thoracic surgery specific purposes. First, they enable phenotyping of baseline functional reserve and perioperative vulnerability. Second, they facilitate monitoring of early postoperative recovery trajectories. Third, they support behavior-change interventions through goal setting, feedback, and remote coaching (2, 3). In broader perioperative literature, pedometer-derived activity shows biologically plausible associations with postoperative outcomes, but thoracic surgery has unique physiologic and symptom-related constraints, including pain-limited breathing, atelectasis risk, chest drains, and dyspnea, which warrant surgery-specific synthesis (4, 5).

Therefore, using a PICOS framework, this systematic review evaluated adult patients undergoing lung cancer surgery or clinically relevant pulmonary resection; wearable-based activity, physiologic, or rehabilitation monitoring as the intervention or exposure; standard perioperative care or non-wearable management as the comparator where available; recovery-related outcomes including length of stay, chest tube duration, postoperative mobility, functional capacity, HRQOL, symptoms, physiologic agreement, and complications; and randomized, nonrandomized, observational, feasibility, or measurement-agreement studies. The aim was to clarify the feasibility, recovery-related findings, and certainty of evidence for wearable-enhanced perioperative care in thoracic surgical oncology.

## 2 Methods

### 2.1 Reporting framework and search strategy

This systematic review was conducted according to the PRISMA 2020 statement (6) and was registered in PROSPERO (CRD420261325339). PubMed, Scopus, and Web of Science Core Collection were searched for English-language full-text studies published between February 2, 1996 and February 2, 2026. The search terms included wearable device, wearable sensor, wearable technology, activity tracker, accelerometer, pedometer, thoracic surgery, lung cancer surgery, lung resection, pulmonary resection, lobectomy, segmentectomy, pneumonectomy, VATS, perioperative monitoring, prehabilitation, and postoperative recovery, together with relevant Boolean combinations. The broad starting year was prespecified in PROSPERO to capture earlier pedometer-based, actigraphy, and research-grade accelerometry studies before consumer-grade wearable devices became widely adopted. No unpublished studies were sought. The complete search strategy is provided in **Supplementary Table 1**.

### 2.2 Inclusion and exclusion criteria

#### 2.2.1 Inclusion criteria

1. Studies involving adult patients undergoing lung cancer surgery or clinically relevant pulmonary resection.
2. Studies evaluating wearable devices or digital monitoring systems used for perioperative activity tracking, physiologic monitoring, sleep monitoring, integration with digital drainage or multimodal systems.
3. Randomized controlled trials (RCTs), prospective cohort studies, observational studies, feasibility studies, or controlled clinical trials conducted in real-world clinical settings.
4. Studies reporting clinical or recovery-related outcomes, including but not limited to length of hospital stay, duration of chest tube placement, postoperative complications, quality of life, rehabilitation or ambulation metrics.
5. Peer-reviewed full-text articles published in English.

#### 2.2.2 Exclusion criteria

1. Studies not involving thoracic surgical populations.
2. Articles focusing exclusively on hardware development, sensor engineering, algorithm development, artificial intelligence model construction without clinical validation in perioperative thoracic surgery patients.
3. Studies lacking objective wearable-derived perioperative data or not reporting clinically meaningful recovery outcomes.
4. Case reports, conference abstracts without full data, editorials, commentaries, white papers, or non-peer-reviewed materials.
5. Studies involving non-surgical lung cancer populations.

### 2.3 PICOS eligibility framework

Eligibility criteria were structured according to the PICOS framework. The population was adult patients undergoing lung cancer surgery or clinically relevant pulmonary resection. The intervention or exposure was perioperative wearable-based activity, physiologic, or rehabilitation monitoring. Comparators included usual care, non-wearable management, traditional drainage, historical controls, or within-person reference measurements where applicable. Outcomes included length of stay, chest tube duration, postoperative mobility, functional capacity, HRQOL, symptoms, physiologic agreement, complications, and feasibility or implementation outcomes. Eligible study designs included randomized trials, nonrandomized trials, prospective observational studies, feasibility studies, and measurement-agreement studies. Prehabilitation was defined as a structured preoperative intervention delivered before surgery to improve functional capacity, physical activity, respiratory reserve, or readiness for postoperative recovery. Wearable-supported prehabilitation referred to programs in which wearable devices were used to monitor activity, provide feedback, support goal setting, or assess adherence before surgery.

### 2.4 Data extraction

Data extraction was performed using a standardized electronic data collection form developed in Microsoft Excel. Two investigators independently extracted study-level data to ensure accuracy and completeness. Extracted variables included bibliographic information, study design, sample size, type of surgery or pulmonary resection, wearable device characteristics, and reported clinical outcomes. Clinical outcomes of interest included length of hospital stay, duration of chest tube placement, postoperative complications, ambulation metrics, sleep duration, health-related quality of life, and patient-reported outcome measures where applicable. Discrepancies in extracted data were resolved through discussion and consensus, and unresolved disagreements were adjudicated by a third reviewer. When multiple reports appeared to originate from the same research program or potentially overlapping cohort, they were treated as companion reports; their findings were extracted separately but were not double-counted as independent evidence in the certainty assessment.

### 2.5 Quality assessment

Methodological quality was independently assessed by two reviewers using design-specific risk-of-bias instruments. Randomized controlled trials were evaluated with the Cochrane Risk of Bias 2 (RoB 2) tool, which assesses bias across five domains including the randomization process, deviations from intended interventions, missing outcome data, outcome measurement, and selective reporting; overall judgments were categorized as low risk, some concerns, or high risk (7). Non-randomized studies were appraised using the Risk of Bias in Non-randomized Studies of Interventions (ROBINS-I) framework, which evaluates bias related to confounding, participant selection, intervention classification, deviations from intended interventions, missing data, outcome measurement, and selective reporting, with overall ratings of low, moderate, serious, or critical risk assigned according to the highest domain-level concern (8). Qualitative certainty judgments for major outcome domains were informed by GRADE principles (9), considering study design, risk of bias, consistency, directness, precision, and whether each outcome reflected clinical effectiveness, feasibility, or measurement agreement. Disagreements were resolved by discussion and, if required, adjudication by a third reviewer. For development, usability, or measurement-agreement studies without comparative clinical-effect endpoints, risk-of-bias judgments were interpreted descriptively, focusing on selection, measurement reliability, missing data, and indirectness rather than causal treatment effects. Detailed domain-level risk-of-bias judgments are provided in **Supplementary Table 2**.

### 2.6 Data synthesis

Given substantial heterogeneity in study design, wearable platforms, monitoring duration, comparator strategies, and outcome definitions, quantitative meta-analysis was not performed. Studies were organized by perioperative phase and major outcome domains, including length of stay, chest tube duration, mobility metrics, functional capacity, health-related quality of life, and physiologic agreement. We extracted and reported study-level numerical results, including risk ratios, mean or median differences, correlation coefficients, regression coefficients, confidence intervals, and *p* values where available. Findings were synthesized narratively by perioperative phase and outcome domain.

## 3 Results

### 3.1 Study selection

**Figure 1:**
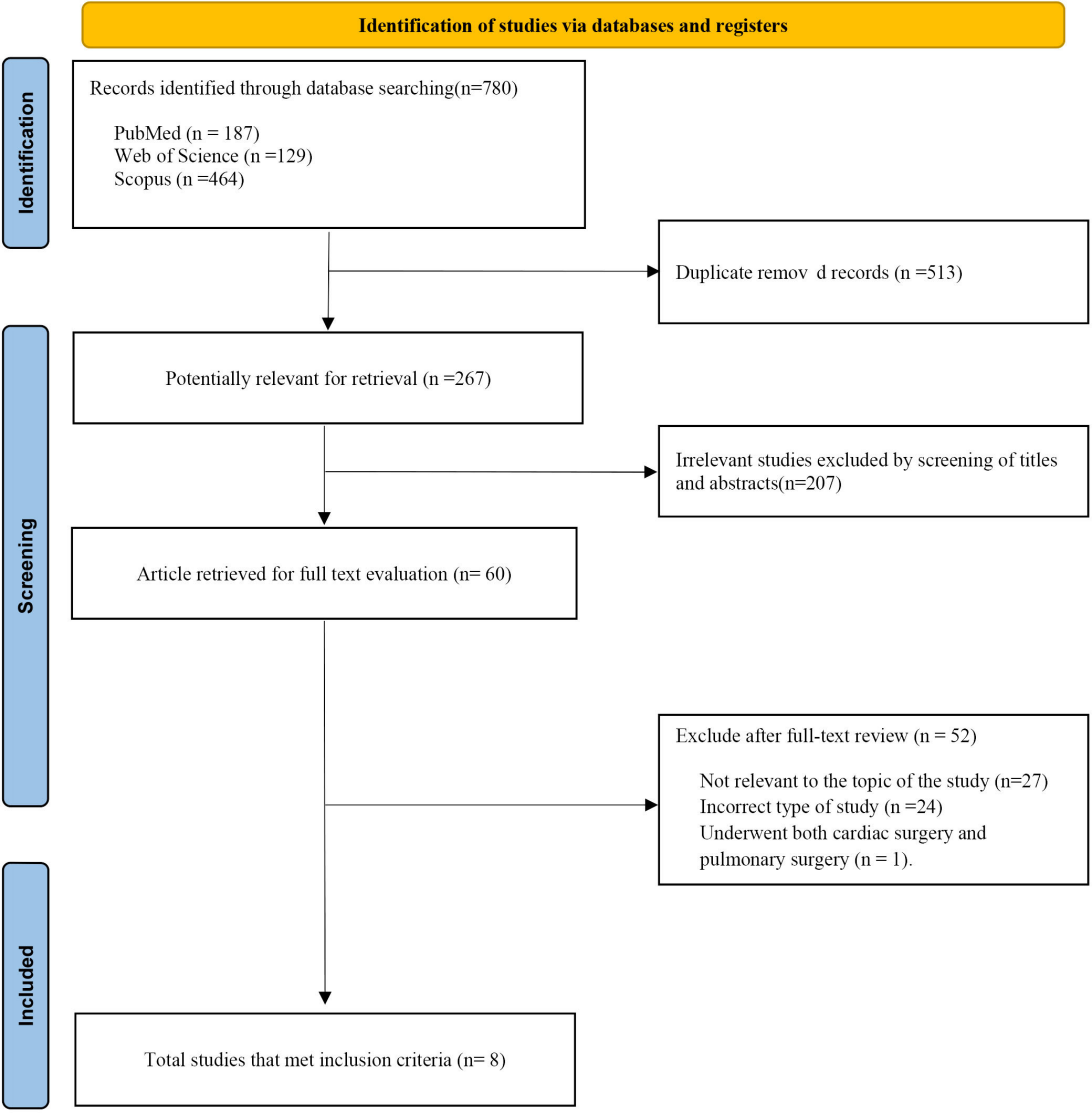
Flow diagram of literature search and study selection according to PRISMA 2020 guidelines.

Across the prespecified search window from February 2, 1996 to February 2, 2026, the database search identified 187 records from PubMed, 464 from Scopus, and 129 from Web of Science Core Collection. After duplicate removal, title/abstract screening, and full-text eligibility assessment, eight reports representing seven independent clinical cohorts were included in the qualitative synthesis. Two reports from the same Dartmouth preoperative exercise/activity-tracker research program were treated as companion reports and were not double-counted as independent evidence in the certainty assessment. Because of substantial heterogeneity in study design, device platform, comparator strategy, monitoring window, and outcome definition, quantitative pooling was not performed. The study selection process is summarized in the PRISMA flow diagram (**Figure 1**).

### 3.2 Study and device characteristics

The eight included reports comprised two randomized trials evaluating perioperative system-level or behavioral interventions (10, 11), one nonrandomized clinical trial with historical controls evaluating staged wearable-guided rehabilitation (12), two prospective observational studies of perioperative mobility or step count (13, 14), two companion single-arm reports evaluating preoperative wearable-supported exercise feasibility and potential effectiveness (15, 16), and one development/usability agreement study integrating smartwatch-derived physiologic data with electronic patient-reported outcomes (ePROs) (17).

The study populations were predominantly adults undergoing lung cancer surgery or clinically relevant pulmonary resection. Device modalities included consumer activity trackers, research-grade accelerometers, wristband step counters, smartwatch-based physiologic monitors, wearable activity/sleep monitors combined with digital chest drainage, and multimodal smartwatch-ePRO systems. The main outcome domains included length of stay, chest tube duration, postoperative mobility, activity intensity, sedentary behavior, sleep, six-minute walk distance (6MWD), health-related quality of life (HRQOL), symptoms, physiologic agreement, and complications. Full study characteristics are summarized in **Table 1**, and detailed study-level outcome extraction is provided in **Supplementary Table 3**.

**Table 1:** Characteristics of included studies.

Study	Design	Population	N	Device/system	Phase	Main endpoint	Key quantitative findings	Evidence role
Patel 2023 (11)	Single-site blinded RCT	Early-stage NSCLC lung resection	102 randomized, 95 analyzed	Fitbit-based Move For Surgery	Preoperative	Prolonged LOS >5 days	Prolonged LOS 7% vs 24%, *p*=0.021	Clinical-effect evidence
Yang 2025 (10)	Open-label single-center RCT	Pulmonary resection, mixed final diagnoses	253	DCD + Amazfit	Postoperative	Chest tube duration, LOS, sleep, ambulation	LOS 77.55 vs 107.02 h, *p*=0.012; chest tube 50.52 vs 73.80 h, *p*=0.028	Indirect lung cancer-specific evidence
Lee 2024 (12)	Nonrandomized clinical trial with historical control	NSCLC curative surgery	194	Fitbit Versa intervention	Pre-op to 6 months	Steps, MVPA, 6MWD, HRQOL	6-month steps 12,321 vs 10,118, *p*=0.007; no 6MWD difference	Hypothesis-generating clinical evidence
Yao 2025 (13)	Prospective observational study	Minimally invasive lung cancer surgery	244	Mi Band 5	Pre-op and POD1–3	LOHS, SF-12, pain	Preop steps vs LOHS *r*=-0.146; POD1 steps vs LOHS *r*=-0.172; POD1 steps vs 1-month PCS *r*=0.186	Association evidence
Finet 2023 (14)	Prospective observational study	Mini-invasive lung surgery	60 enrolled, 56 valid	ActiGraph GT3X	POD1–5	Steps, light activity, sedentary bouts	Daily steps stable; light activity increased; prolonged sedentary bouts decreased	Mobility-metric evidence
Finley 2020 (15)	Single-arm feasibility study	Stage I–III lung cancer surgery candidates	30 enrolled	Garmin Vivoactive HR	Preoperative	Feasibility, sync, acceptability	79% completed pre-op activities; 71% synced device; data on 70% of pre-op days	Feasibility evidence
Finley 2021 (16)	Companion proof-of-concept report	Same/related pre-op lung cancer cohort	18 with device data	Garmin Vivoactive HR	Preoperative	MVPA, 6MWD	MVPA 20.4 min/day; 6MWD +13.8 m, *p*=0.14; 47% achieved ≥14 m improvement	Companion outcome report
Wang 2025 (17)	Development and usability/agreement study	NSCLC thoracic surgery	288	HUAWEI WATCH D + ePRO	In-hospital	Agreement with EHR, outlier detection	Temperature bias 0.02°C; HR bias 0.26 bpm; SpO_2_ bias -0.06%	Measurement-feasibility evidence

Eight reports representing seven independent clinical cohorts were included. Finley et al., 2020 and Finley et al., 2021 were treated as companion reports from the same Dartmouth preoperative wearable-supported exercise research program and were not double-counted as independent evidence in the certainty assessment. Yang et al., 2025 included a mixed pulmonary resection population rather than a lung cancer-only cohort and was therefore interpreted as indirect evidence for lung cancer-specific conclusions. Wang et al., 2025 was interpreted as measurement-feasibility evidence because it evaluated smartwatch-ePRO integration and agreement with electronic health record measurements rather than a comparative clinical-effect endpoint.

DCD, digital chest drainage; EHR, electronic health record; ePRO, electronic patient-reported outcome; HR, heart rate; HRQOL, health-related quality of life; LOS, length of stay; LOHS, length of hospital stay; MVPA, moderate-to-vigorous physical activity; NSCLC, non-small cell lung cancer; PCS, physical component score; POD, postoperative day; RCT, randomized controlled trial; SpO_2_, peripheral oxygen saturation; 6MWD, six-minute walk distance.

### 3.3 Risk of bias and certainty context

The two randomized trials were judged as having “some concerns” using RoB 2, mainly because of limited blinding in behavioral or device-based interventions and potential performance bias. However, their principal endpoints, including prolonged length of stay, chest tube duration, and postoperative hospital stay, were objective and therefore less susceptible to subjective outcome-measurement bias. The nonrandomized clinical trial with historical controls was rated as serious risk of bias due to confounding and possible time-period effects. Prospective observational studies were considered at moderate risk of bias because of residual confounding, reverse causality, and incomplete control of perioperative factors. Single-arm feasibility studies were rated as serious risk of bias for causal inference because they lacked comparator groups and had small sample sizes. The development/usability study was not designed to estimate a comparative clinical treatment effect and was therefore interpreted as measurement-feasibility evidence rather than intervention-effect evidence.

Overall, certainty was highest for selected objective in-hospital endpoints supported by randomized evidence, but remained limited by single-center design, intervention heterogeneity, and indirectness. Evidence for observational associations, long-term functional recovery, HRQOL, and complications was generally low or very low certainty. The study-level risk-of-bias assessment is summarized in **Table 2**. Outcome-level certainty judgments are summarized in **Table 3**, with detailed GRADE-informed explanations provided in **Supplementary Table 4**.

**Table 2:** Risk of bias assessment summary.

Study	Study design	Assessment tool	Overall risk of bias	Key concerns	Evidence interpretation
Patel et al., 2023 (11)	Randomized controlled trial	RoB 2	Some concerns	Behavioral intervention; limited participant blinding; single-center design; multicomponent preconditioning program	Clinical-effect evidence for selected objective endpoints, especially prolonged length of stay
Yang et al., 2025 (10)	Open-label randomized controlled trial	RoB 2	Some concerns	Open-label design; potential performance bias; mixed pulmonary resection population rather than lung cancer-only cohort	Indirect lung cancer-specific evidence for chest tube duration, length of stay, sleep, and ambulation
Lee et al., 2024 (12)	Nonrandomized clinical trial with historical controls	ROBINS-I	Serious	Historical control group; potential confounding; time-period effects; residual differences in perioperative care	Hypothesis-generating clinical evidence for longer-term wearable-guided rehabilitation
Yao et al., 2025 (13)	Prospective observational cohort	ROBINS-I	Moderate	Observational design; residual confounding; reverse causality; no wearable-driven intervention	Association evidence linking perioperative step count with length of stay and physical recovery
Finet et al., 2023 (14)	Prospective observational accelerometry study	ROBINS-I	Moderate	No control group; short monitoring window; mobility metrics heterogeneous; limited clinical outcome linkage	Mobility-metric evidence characterizing early postoperative activity and sedentary behavior
Finley et al., 2020 (15)	Single-arm feasibility study	ROBINS-I/descriptive appraisal	Serious for causal inference	No comparator group; small sample size; feasibility-focused design	Feasibility evidence only; not designed to estimate clinical effectiveness
Finley et al., 2021 (16)	Companion single-arm proof-of-concept report	ROBINS-I/descriptive appraisal	Serious for causal inference	No comparator group; small sample size; potential overlap with the same Dartmouth preoperative exercise program	Companion feasibility/outcome evidence for MVPA and 6MWD; not independent confirmatory evidence
Wang et al., 2025 (17)	Development and usability/measurement-agreement study	Descriptive appraisal	Not a causal-effect study	No comparative clinical-effect endpoint; no escalation algorithm tested; indirectness for clinical benefit	Measurement-feasibility evidence only for smartwatch-ePRO integration and physiologic agreement

RoB 2 was used for randomized trials, and ROBINS-I was used for nonrandomized comparative or observational studies. For single-arm feasibility studies and measurement-agreement studies, the judgment reflects risk of bias for clinical causal inference, not feasibility or measurement performance alone. Wang et al. was interpreted descriptively because it was not designed to estimate a comparative treatment effect.

ePRO, electronic patient-reported outcome; MVPA, moderate-to-vigorous physical activity; RoB 2, Cochrane Risk of Bias 2 tool; ROBINS-I, Risk Of Bias In Non-randomized Studies of Interventions; 6MWD, six-minute walk distance.

**Table 3:** Summary of findings by outcome domain and qualitative certainty of evidence.

Outcome domain	Main supporting evidence	Key quantitative findings	Qualitative certainty	Interpretation
Prolonged length of stay	Patel et al., 2023 (11)	Prolonged hospital stay >5 days occurred in 7% versus 24% of patients in the intervention and control groups, respectively (3/45 vs 12/50; *p*=0.021). Mean hospital stay was also shorter in the intervention group (2.67 vs 4.44 days; mean difference, -1.77 days; 95% CI, -2.90 to -0.65; *p*=0.002).	Moderate	Randomized evidence suggests a potential reduction in prolonged hospitalization, but the intervention was multicomponent and the wearable-specific contribution cannot be isolated.
Postoperative hospital stay and chest tube duration	Yang et al., 2025 (10); Patel et al., 2023 (11)	In the digital chest drainage RCT, postoperative hospital stay was shorter in the DCD group than in the TCD group (77.55 ± 32.89 vs 107.02 ± 124.87 h; *p*=0.012), and chest tube duration was shorter (50.52 ± 28.73 vs 73.80 ± 115.90 h; *p*=0.028). In Patel et al., chest tube duration was numerically shorter but not statistically significant (2.93 vs 5.46 days; *p*=0.076).	Low-to-moderate	Objective in-hospital endpoints showed favorable signals, but certainty was limited by open-label design, indirectness of the mixed pulmonary resection cohort, and inconsistent statistical significance.
Perioperative step count and length of stay	Yao et al., 2025 (13)	Preoperative and POD1 step counts were weakly but significantly correlated with shorter hospital stay (r=-0.146, *p*=0.023; r=-0.172, *p*=0.018). POD1 step count was also associated with 1-month SF-12 physical component recovery (*r*=0.186, *p*=0.013).	Low	Step count may serve as an objective recovery marker, but associations were modest and observational; reverse causality and residual confounding remain likely.
Postoperative mobility, activity intensity, and sedentary behavior	Finet et al., 2023 (14); Lee et al., 2024 (12); Yao et al., 2025 (13); Yang et al., 2025 (10)	Finet et al. found that daily steps and mean cadence did not significantly change during POD1–4, whereas light-intensity activity increased and prolonged sedentary bouts decreased. Lee et al. reported higher 6-month daily steps in the intervention group than controls (12,321 vs 10,118; *p*=0.007).	Low	Wearables can characterize mobility recovery, but outcomes, devices, monitoring windows, and intervention contexts were heterogeneous. Step count alone may be insufficient to capture early postoperative recovery.
Functional capacity and patient-reported recovery	Finley et al., 2021 (16); Lee et al., 2024 (12); Yao et al., 2025 (13)	Finley et al. reported a non-significant 6MWD increase of 13.8 m (*p*=0.14), although 47% achieved ≥14 m improvement. Lee et al. found no significant between-group difference in 6MWD at 6 months despite higher free-living activity. Pain was associated with worse SF-12 physical recovery at 1 and 3 months in Yao et al.	Low	Wearable-derived free-living activity and clinic-based functional tests appear to capture different dimensions of recovery. Symptom burden should be interpreted alongside wearable metrics.
Physiologic monitoring and measurement agreement	Wang et al., 2025 (17)	Smartwatch-derived measurements showed close agreement with electronic health record values, with biases of 0.02°C for body temperature, 0.26 bpm for heart rate, and -0.06% for oxygen saturation.	Moderate for measurement feasibility; low for clinical benefit	Continuous physiologic monitoring appears feasible, but no study has shown that wearable-derived alerts or escalation algorithms improve clinical outcomes.
Feasibility and implementation	Finley et al., 2020 (15); Patel et al., 2023 (11); Lee et al., 2024 (12); Finet et al., 2023 (14); Wang et al., 2025 (17)	Finley et al. reported 79% completion of preoperative activities, 71% device synchronization, and data transmission on 70% of preoperative days. Patel et al. reported Fitbit wear for 94.4% of trial time. Lee et al. reported 95% device compliance.	Low-to-moderate	Feasibility findings were generally favorable, but most studies were single-center and involved selected patients willing or able to use digital devices.
Complications and safety endpoints	Patel et al., 2023 (11); Yang et al., 2025 (10); Yao et al., 2025 (13)	Patel et al. found no significant differences in intraoperative, in-hospital, or postdischarge adverse events. Yang et al. found no significant difference in postoperative pulmonary complications (7.8% vs 4.0%; *p*=0.157). Yao et al. reported complications descriptively, including 18/244 total complications and 10/244 pulmonary complications.	Very low	Current evidence is insufficient to determine whether wearable-enhanced perioperative pathways reduce complications or safety events.

Qualitative certainty was judged narratively using GRADE-informed principles, considering study design, risk of bias, inconsistency, indirectness, imprecision, and whether the outcome represented clinical effectiveness, feasibility, or measurement agreement. Because quantitative pooling was not performed, certainty was assessed at the outcome-domain level rather than through formal meta-analytic GRADE. Detailed domain-level explanations are provided in **Supplementary Table 4**. DCD, digital chest drainage; EHR, electronic health record; POD, postoperative day; RCT, randomized controlled trial; SF-12, 12-Item Short Form Health Survey; TCD, traditional chest drainage; 6MWD, six-minute walk distance.

### 3.4 Length of stay and chest tube duration

The most direct randomized evidence for length of stay came from the Move For Surgery trial (11). In this single-site blinded randomized trial, prolonged hospital stay greater than 5 days occurred in 3 of 45 patients (7%) in the wearable-enhanced preconditioning group and 12 of 50 patients (24%) in the usual-care group (*p*=0.021). Mean hospital stay was also shorter in the intervention group than in controls (2.67 vs 4.44 days; mean difference, -1.77 days; 95% CI, -2.90 to -0.65; *p*=0.002) *(*11). Chest tube duration was numerically shorter in the intervention group but did not reach statistical significance (2.93 vs 5.46 days; *p*=0.076) *(*11). These findings suggest a potential benefit for wearable-supported preconditioning on objective hospitalization endpoints, although the intervention included multiple components beyond the wearable device alone.

A second randomized trial evaluated digital chest drainage combined with wearable activity/sleep monitoring after pulmonary resection (10). Compared with traditional chest drainage, the digital chest drainage group had shorter postoperative hospital stay (77.55 ± 32.89 vs 107.02 ± 124.87 hours; *p*=0.012) and shorter chest tube duration (50.52 ± 28.73 vs 73.80 ± 115.90 hours; *p*=0.028) *(*10). The same study reported fewer postoperative chest radiographs, but no significant difference in postoperative pulmonary complications (7.8% vs 4.0%; *p*=0.157) *(*10). Because this trial enrolled a mixed pulmonary resection population and was not restricted to lung cancer, its applicability to lung cancer-specific conclusions was considered indirect.

In the prospective observational cohort by Yao et al., higher preoperative and postoperative day 1 step counts were weakly but significantly correlated with shorter length of hospital stay (*r*=-0.146, *p*=0.023; *r*=-0.172, *p*=0.018) *(*13). Multivariable analysis identified postoperative day 1 step count as an independent predictor of length of hospital stay (13). These findings support step count as a candidate recovery marker, but the observational design precludes causal interpretation.

### 3.5 Postoperative mobility, activity intensity, and sedentary behavior

Evidence on postoperative mobility indicated that recovery after lung surgery is multidimensional and not fully captured by step count alone. In a prospective observational accelerometry study of minimally invasive lung surgery, 60 patients were enrolled and 56 provided at least one valid day of ActiGraph GT3X data (14). Daily step counts and mean cadence did not significantly change during the first four postoperative days, whereas total activity counts, counts per minute, peak one-minute cadence, and light-intensity physical activity increased over time (14). Sedentary behavior also improved, with fewer prolonged sedentary bouts of at least 60 consecutive minutes and more short sedentary bouts (14). These findings suggest that activity intensity and sedentary-bout structure may be more sensitive than step count alone for characterizing early postoperative recovery.

In the Yao et al. cohort of 244 patients undergoing minimally invasive lung cancer surgery, all patients completed preoperative step monitoring. Median preoperative daily step count was 7,233 steps, whereas median postoperative step count was markedly lower on postoperative day 1 (83 steps), then increased on postoperative days 2 and 3 (820 and 1,151 steps, respectively) (13). Postoperative day 1 step count was correlated with 1-month change in SF-12 physical component score (*r*=0.186, *p*=0.013). Step counts on postoperative days 2 and 3 were also associated with 3-month physical recovery in supplementary analyses, but the effect sizes were modest and vulnerable to confounding.

Longer-term mobility was evaluated in the nonrandomized clinical trial by Lee et al. (12). The intervention group received a staged personalized exercise program monitored with a wearable device from the preoperative period to 6 months after surgery, whereas the control group was drawn from a historical cohort. At 2 weeks after surgery, both groups showed a decrease in daily steps, but the decrease was smaller in the intervention group. At 6 months, the intervention group had higher daily steps than controls (12,321 [95% CI, 8,749 to 15,761] vs 10,118 [95% CI, 7,341 to 13,420]; *p*=0.007) and greater vigorous physical activity (33.6 vs 18.5 minutes; *p*=0.003) *(*12). These findings suggest improved free-living activity, but the historical-control design limits causal certainty.

### 3.6 Sleep and physiologic recovery monitoring

Sleep was mainly reported in the digital drainage randomized trial. Patients in the digital chest drainage group had longer postoperative sleep duration during the early postoperative period and higher postoperative walking steps, particularly on postoperative days 2 and 3 (10). However, because sleep and ambulation may be influenced by chest tube discomfort, nursing practice, analgesia, and patient selection, these findings were interpreted as supportive but not definitive evidence for wearable-enhanced recovery monitoring.

A multimodal development and usability study integrated smartwatch-derived physiologic monitoring with ePROs in 288 patients with non-small cell lung cancer undergoing thoracic surgery (17). Bland-Altman analysis showed close agreement between wearable-derived and electronic health record measurements for body temperature, heart rate, and oxygen saturation. The reported biases were 0.02°C for body temperature, 0.26 beats per minute for heart rate, and -0.06% for oxygen saturation (17). Wearable monitoring also captured a substantially larger number of physiologic measurements and outlier events than episodic ward-based recording. These results support measurement feasibility and workflow potential, but the study did not test whether continuous monitoring improved clinical outcomes.

### 3.7 Functional capacity, HRQOL, symptoms, and pain

Functional capacity was assessed primarily using 6MWD. In the Finley et al. proof-of-concept report, 18 patients with device data participated in a surgeon-delivered exercise prescription supported by a Garmin Vivoactive HR tracker (16). Mean moderate-to-vigorous physical activity was 20.4 minutes per day, and the target of 30 minutes per day was achieved on 16.4% of preoperative days (16). Mean 6MWD increased from 456.7 m at baseline to 471.1 m on the day of surgery, corresponding to a mean improvement of 13.8 m, but this change was not statistically significant (*p*=0.14) (16). Eight of 17 participants (47%) achieved a clinically meaningful improvement of at least 14 m (16). Because this was a small single-arm analysis, it provides feasibility and hypothesis-generating evidence rather than proof of effectiveness.

In the Lee et al. nonrandomized clinical trial, no significant between-group difference was observed in 6MWD at 6 months after surgery despite greater improvement in free-living daily steps and vigorous physical activity in the wearable-intervention group (12). The intervention group reported better physical function, less dyspnea, and less pain at 2 weeks, and less dyspnea at 6 months, compared with controls (12). These findings indicate that wearable-guided rehabilitation may influence free-living activity and patient-reported symptoms, but not necessarily clinic-based walking capacity.

In the Yao et al. observational study, SF-12 physical component score declined at 1 month after surgery and partially recovered at 3 months, but remained lower than baseline (13). Pain was associated with worse physical recovery, significantly affecting SF-12 physical component score changes at 1 month and 3 months (13). Specifically, pain was reported as a significant predictor of physical component score change at 1 month (*β*=-3.33, *p*<0.001 in the abstract; *β*=-3.70, *p*<0.001 in the regression table) and 3 months (*β*=-3.06, *p*<0.001) (13). These results reinforce the importance of symptom burden when interpreting wearable-derived mobility metrics.

### 3.8 Feasibility, implementation, and safety endpoints

Feasibility evidence was strongest for device use, synchronization, and acceptability, rather than clinical effectiveness. In the Finley et al. feasibility study, 30 patients scheduled for stage I–III lung cancer surgery received a Garmin Vivoactive HR and a preoperative exercise prescription (15). Seventy-nine percent completed preoperative study activities, 71% successfully synchronized the device during the preoperative period, and wearable data were transmitted on an average of 70% of preoperative days (15). In the Move For Surgery trial, patients in the intervention arm wore the Fitbit for a mean of 94.4% of trial time, and 96% of surveyed patients stated that they would continue using the activity tracker or related lifestyle changes after the trial (11). In the Lee et al. study, compliance with wearable device use was reported as 95% (12). In the Finet et al. accelerometry study, acceptability of wearing the accelerometer was excellent, with a median score of 10 on a 10-point scale (14).

Evidence for complications was sparse and underpowered. In the Move For Surgery trial, there were no statistically significant differences in intraoperative complications, adverse events during hospitalization, or postdischarge adverse events at 3 or 12 weeks (11). In the digital drainage randomized trial, postoperative pulmonary complications did not differ significantly between digital and traditional drainage groups (10). In the Yao et al. cohort, postoperative complications occurred in 18 patients (7.4%), including pulmonary complications in 10 patients (4.1%), but the study primarily evaluated associations between step count and recovery rather than complication reduction (13). Overall, the available evidence was insufficient to determine whether wearable-enhanced perioperative pathways reduce postoperative complications or safety events.

## 4 Discussion

### 4.1 Principal findings

This systematic review synthesized eight reports representing seven independent clinical cohorts evaluating wearable or wearable-enabled perioperative monitoring in lung cancer surgery or clinically relevant pulmonary resection. Overall, the available evidence suggests that wearable-based monitoring is feasible and can provide objective recovery-related metrics, including step count, activity intensity, sedentary behavior, sleep, heart rate, oxygen saturation, and patient-reported symptoms.

The strongest clinical-effect evidence came from two randomized studies. The Move For Surgery trial suggested that wearable-enhanced preconditioning was associated with a lower incidence of prolonged hospital stay after lung cancer surgery (11). The digital chest drainage trial suggested shorter chest tube duration and postoperative hospital stay when digital drainage was combined with wearable monitoring of ambulation and sleep (10). Nevertheless, both studies evaluated multicomponent perioperative pathways rather than wearable devices as isolated interventions. Observational and feasibility studies further supported the potential value of wearable-derived recovery markers, but these findings were mainly exploratory and vulnerable to confounding, reverse causality, and selection bias.

Thus, the central finding of this review is not that wearables are already proven to improve postoperative outcomes, but rather that they represent a feasible objective measurement layer within thoracic perioperative care. Current evidence supports their further evaluation as tools for recovery phenotyping and hypothesis generation, while definitive evidence for routine clinical implementation remains insufficient.

### 4.2 Interpretation by outcome domain

For length of stay, the most clinically relevant signal came from the Move For Surgery randomized trial. In that study, prolonged hospitalization greater than 5 days occurred less frequently in the intervention group than in the control group (11). This endpoint is objective and clinically meaningful, and therefore provides moderate-certainty evidence for a potential benefit of wearable-enabled preconditioning. However, the intervention included several components, including exercise prescription, deep breathing exercises, nutrition advice, sleep hygiene, smoking cessation education, remote encouragement, and Fitbit-guided goal setting. Therefore, the observed reduction in prolonged hospital stay cannot be attributed to the wearable device alone.

Chest tube duration and postoperative hospital stay were also improved in the digital chest drainage randomized study (10). This finding is relevant to thoracic surgery because chest tube management directly affects pain, ambulation, sleep, discharge readiness, and postoperative imaging use. However, this trial enrolled a mixed pulmonary resection population and was not restricted to lung cancer patients. In addition, the core intervention was a digital drainage system, with wearable activity and sleep monitoring used as part of the broader postoperative assessment. Therefore, this evidence should be interpreted as indirect support for wearable-enabled thoracic recovery pathways rather than direct evidence that wearable devices independently shorten chest tube duration. This finding is also consistent with previous thoracic surgical literature suggesting that digital chest drainage may facilitate more objective chest tube management and support discharge decision-making after pulmonary resection (18, 19). However, in the present review, the digital drainage evidence should be interpreted as contextual support rather than direct evidence of a wearable-specific effect.

For postoperative mobility, the included studies consistently showed that activity data can be captured in the perioperative period, but the most informative metric may not be step count alone. Finet et al. demonstrated that daily steps and mean cadence did not significantly change during the first postoperative days, whereas light-intensity activity increased and sedentary-bout patterns improved (14). This finding is important because patients after lung surgery may have pain-limited breathing, chest tube-related discomfort, and restricted ward mobility, all of which may alter movement patterns without producing large changes in total daily steps. Composite mobility metrics incorporating light activity, sedentary interruption, cadence, and step recovery may therefore be more sensitive than step count alone.

The prospective cohort by Yao et al. further supports step count as a candidate recovery marker. Preoperative and postoperative day 1 step counts were weakly but significantly associated with length of hospital stay, and postoperative day 1 steps were associated with short-term physical recovery (13). However, the correlations were modest, and patients who recover faster may naturally walk more. Therefore, step count should be interpreted as a recovery signal rather than a proven modifiable causal factor.

### 4.3 Functional recovery, symptoms, and patient-centered outcomes

Evidence for functional capacity and patient-centered recovery was less consistent than evidence for objective in-hospital endpoints. In the Finley proof-of-concept study, 6-minute walk distance improved numerically after a surgeon-delivered exercise prescription supported by an activity tracker, but the mean change was not statistically significant (16). In the Lee et al. nonrandomized clinical trial, the wearable-guided intervention improved free-living activity at 6 months, including daily steps and vigorous physical activity, but did not produce a significant between-group difference in 6-minute walk distance (12). These findings suggest that wearable-derived free-living activity and clinic-based functional tests may capture different dimensions of recovery.

This distinction is clinically important. A patient may increase daily activity or reduce sedentary time without showing a parallel improvement in 6-minute walk distance. Conversely, a standardized 6-minute walk distance assessment may not fully represent the patient’s actual recovery behavior at home. Therefore, future studies should avoid treating wearable-derived activity and traditional functional capacity tests as interchangeable outcomes. Instead, they should define how each metric contributes to perioperative recovery assessment. These findings are compatible with the broader lung cancer rehabilitation literature, in which structured preoperative or postoperative rehabilitation may improve selected functional outcomes, but effects vary according to intervention intensity, adherence, timing, and outcome definition (20, 21).

Patient-reported symptoms also need to be integrated into interpretation of wearable data. Yao et al. showed that pain was significantly associated with worse SF-12 physical recovery at 1 and 3 months (13). Wang et al. demonstrated that smartwatch-derived physiologic data could be combined with ePROs to characterize postoperative symptom trajectories (17). These findings suggest that wearable metrics should not be interpreted in isolation. Low activity may reflect poor cardiopulmonary reserve, pain, sleep disruption, dyspnea, anxiety, chest tube discomfort, or postoperative complications. Multimodal systems combining activity, vital signs, and patient-reported symptoms may therefore provide a more clinically interpretable recovery profile than activity tracking alone.

### 4.4 Wearable-specific effects versus multimodal perioperative interventions

A key interpretive challenge is that most included studies did not evaluate wearable devices as standalone interventions. Instead, wearables were embedded within broader perioperative pathways, including prehabilitation programs, remote coaching, digital chest drainage, ePRO capture, and ERAS-based postoperative care. This distinction is critical. This interpretation is aligned with ERAS/ESTS recommendations for enhanced recovery after lung surgery, which emphasize multimodal perioperative care, early mobilization, optimized analgesia, respiratory care, chest tube management, and discharge readiness (1).

The available evidence is best interpreted as evidence for wearable-enabled perioperative pathways rather than evidence for wearable devices alone. Wearables may contribute by improving feedback, adherence monitoring, goal setting, early detection of abnormal recovery, and objective documentation of mobilization. However, whether these device-derived data directly change clinical decisions or outcomes remains largely unproven. For example, a wearable may record low step counts or abnormal oxygen saturation, but clinical benefit depends on whether the care team receives the information, interprets it correctly, acts on it promptly, and avoids unnecessary alarms or interventions.

Therefore, future trials should prespecify whether the wearable is intended as a monitoring tool, a behavioral intervention, a clinical decision-support trigger, or part of a multimodal rehabilitation program. Without this distinction, it will remain difficult to isolate the active component of wearable-enhanced care.

### 4.5 Thoracic surgery-specific implications

Thoracic surgery has several features that make wearable-based monitoring particularly relevant but also difficult to interpret. Lung resection involves single-lung ventilation, postoperative pain, impaired cough, atelectasis risk, air leak, pneumothorax risk, chest tube management, and postoperative pulmonary complications. These factors can influence activity, sleep, oxygen saturation, respiratory symptoms, and discharge readiness. Therefore, evidence from general surgery or abdominal surgery should be used only as contextual background and not as direct evidence for thoracic oncology.

Within thoracic ERAS pathways, wearables may be most useful as an objective measurement layer. They can quantify whether early mobilization targets are actually achieved, describe recovery trajectories after chest tube removal, identify patients with delayed return to baseline activity, and support post-discharge rehabilitation. However, thresholds for clinical action remain undefined. For instance, it is unclear what degree of step-count reduction, persistent tachycardia, oxygen desaturation, sleep disruption, or symptom escalation should trigger nursing review, imaging, medication adjustment, or hospital readmission evaluation.

**Figure 2:**
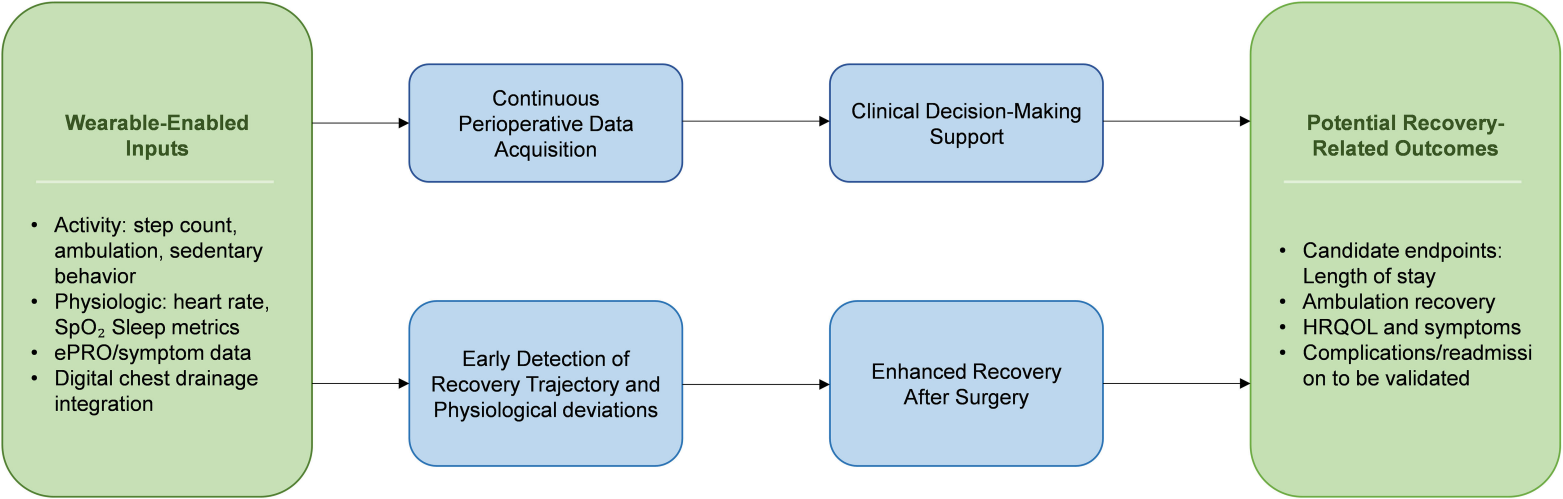
Conceptual framework of wearable-based perioperative monitoring within thoracic ERAS pathways. Arrows indicate hypothesized data flow and potential clinical pathways rather than proven causal effects.

The Wang et al. study provides encouraging measurement-feasibility evidence by showing close agreement between wearable-derived and electronic health record measurements for body temperature, heart rate, and oxygen saturation (17). However, measurement agreement is not the same as clinical effectiveness. Future work must determine whether continuous monitoring improves outcomes, reduces complications, shortens hospital stay, or improves patient experience without increasing alarm burden, workload, or unnecessary investigations. This conceptual distinction is summarized in **Figure 2**.

### 4.6 Future research priorities

Future studies should move from feasibility and association toward clinically actionable evaluation. First, multicenter randomized trials are needed to test wearable-enabled pathways with prespecified clinical endpoints, such as postoperative pulmonary complications, length of stay, readmission, delayed functional recovery, and patient-reported recovery. Second, wearable-derived endpoints should be standardized. At minimum, future trials should clearly define wear time, valid monitoring days, step-count thresholds, activity intensity categories, sedentary-bout metrics, missing-data handling, and timing of postoperative assessment.

Third, studies should distinguish wearable-specific effects from the effects of broader perioperative interventions. Factorial or pragmatic trial designs may help determine whether feedback, remote coaching, automated alerts, or clinician-facing dashboards add benefit beyond simple passive monitoring. Fourth, thoracic-specific escalation algorithms are needed. These algorithms should incorporate activity, oxygen saturation, heart rate, sleep, pain, dyspnea, chest tube status, and patient-reported symptoms rather than relying on a single metric.

Finally, implementation outcomes should be evaluated alongside clinical outcomes. These include patient adherence, usability, data completeness, clinician workload, alarm fatigue, privacy and data security, cost-effectiveness, and equity of access. Without these data, wearable technologies may remain promising measurement tools without a clearly defined role in routine thoracic surgical care. Future trials should also follow digital health reporting standards and evaluate implementation outcomes, including usability, data completeness, workflow integration, alarm burden, privacy, and cost-effectiveness (22–24).

## 5 Limitations

Several limitations should be acknowledged. The evidence base was small and heterogeneous, with substantial variation in study design, device platform, monitoring window, intervention content, comparator strategy, and outcome definition, which precluded quantitative meta-analysis. Causal inference was limited because wearables were often embedded within multicomponent perioperative pathways, making it difficult to isolate their independent effect. Generalizability may also be limited, as most studies were single-center and enrolled selected patients willing or able to use digital devices. Finally, clinically important outcomes, including complications, readmission, long-term recovery, cost-effectiveness, escalation thresholds, and implementation burden, were inconsistently reported or underpowered.

## 6 Conclusion

Wearable-based perioperative monitoring appears feasible and may provide objective recovery signals in lung cancer surgery, including activity, sedentary behavior, sleep, and selected physiologic parameters. Current evidence suggests that these metrics may help characterize perioperative recovery trajectories, but remains insufficient to confirm that wearable monitoring itself improves clinical outcomes. Wearables should therefore be viewed as an evolving measurement layer within structured thoracic perioperative pathways rather than standalone interventions. Future studies should determine how wearable-derived signals can be standardized, clinically interpreted, and translated into actionable decisions that improve patient-centered recovery.

## Data Availability

The original contributions presented in the study are included in the article/**Supplementary Material**. Further inquiries can be directed to the corresponding author.
