# Supplementary material for: Wearable technologies for perioperative recovery monitoring in lung cancer surgery: a systematic review of feasibility, recovery outcomes, and evidence certainty
Source: Front Oncol. 2026 Jun 3;16:1832349. doi: 10.3389/fonc.2026.1832349 (PMC13271973; doi:10.3389/fonc.2026.1832349)
Supplement: Supplementary file 1 [file Table1.docx]

Supplementary Material

Table S1. Detailed database search strategy

Table S2. Domain-level risk-of-bias assessment

Table S3. Detailed outcome extraction from included reports

Table S4. Outcome-level qualitative certainty assessment

Supplementary Table S1. Detailed database search strategy

| Database | Search date | Publication window | Records retrieved | Search field | Full search strategy |
| --- | --- | --- | --- | --- | --- |
| PubMed | February 2, 2026 | February 2, 1996 to February 2, 2026 | 187 | Title/Abstract | ((wearable device OR wearable devices OR wearable* OR wearable sensor OR wearable sensors OR smartwatch* OR smart watch OR smart watches OR activity tracker OR activity trackers OR fitness tracker OR fitness trackers OR pedometer* OR accelerometer* OR actigraph* OR actigraphy OR digital health OR mHealth OR remote monitoring OR continuous monitoring) AND (lung cancer OR lung neoplasm OR lung neoplasms OR NSCLC OR thoracic surgery OR lung surgery OR pulmonary resection OR lung resection OR lobectomy OR segmentectomy OR wedge resection OR pneumonectomy OR VATS OR RATS) AND (perioperative OR preoperative OR postoperative OR prehabilitation OR rehabilitation OR recovery OR ambulation OR length of stay OR chest tube OR chest drainage OR digital drainage)) |
| Scopus | February 2, 2026 | February 2, 1996 to February 2, 2026 | 464 | Title/Abstract/Keywords | TITLE-ABS-KEY((wearable device OR wearable devices OR wearable* OR wearable sensor OR wearable sensors OR smartwatch* OR "smart watch" OR "smart watches" OR "activity tracker" OR "fitness tracker" OR pedometer* OR accelerometer* OR actigraph* OR actigraphy OR "digital health" OR mHealth OR "remote monitoring" OR "continuous monitoring") AND ("lung cancer" OR "lung neoplasm" OR NSCLC OR "thoracic surgery" OR "lung surgery" OR "pulmonary resection" OR "lung resection" OR lobectomy OR segmentectomy OR "wedge resection" OR pneumonectomy OR VATS OR RATS) AND (perioperative OR preoperative OR postoperative OR prehabilitation OR rehabilitation OR recovery OR ambulation OR "length of stay" OR "chest tube" OR "chest drainage" OR "digital drainage")) |
| Web of Science Core Collection | February 2, 2026 | February 2, 1996 to February 2, 2026 | 129 | Topic (TS) | TS=((wearable device OR wearable devices OR wearable* OR wearable sensor OR wearable sensors OR smartwatch* OR "smart watch" OR "smart watches" OR "activity tracker" OR "fitness tracker" OR pedometer* OR accelerometer* OR actigraph* OR actigraphy OR "digital health" OR mHealth OR "remote monitoring" OR "continuous monitoring") AND ("lung cancer" OR "lung neoplasm" OR NSCLC OR "thoracic surgery" OR "lung surgery" OR "pulmonary resection" OR "lung resection" OR lobectomy OR segmentectomy OR "wedge resection" OR pneumonectomy OR VATS OR RATS) AND (perioperative OR preoperative OR postoperative OR prehabilitation OR rehabilitation OR recovery OR ambulation OR "length of stay" OR "chest tube" OR "chest drainage" OR "digital drainage")) |

**Note:** The broad starting date was prespecified to capture earlier pedometer-based, actigraphy, and research-grade accelerometry studies before consumer-grade wearable devices became widely adopted. No unpublished studies were sought.

Supplementary Table S2A. Domain-level risk-of-bias assessment for randomized trials (RoB 2)

| Study | Randomization process | Deviations from intended interventions | Missing outcome data | Measurement of outcome | Selection of reported results | Overall judgment |
| --- | --- | --- | --- | --- | --- | --- |
| Yang et al., 2025 [10] | Low | Some concerns | Low | Low | Low | Some concerns |
| Patel et al., 2023 [11] | Low | Some concerns | Low | Low | Low | Some concerns |

Supplementary Table S2B. Domain-level risk-of-bias assessment for nonrandomized, observational, and single-arm studies

| Study | Confounding | Selection of participants | Classification of exposure/intervention | Deviations from intended exposure/intervention | Missing data | Outcome measurement | Selection of reported results | Overall risk | Evidence interpretation |
| --- | --- | --- | --- | --- | --- | --- | --- | --- | --- |
| Lee et al., 2024 [12] | Serious | Moderate | Moderate | Moderate | Low | Moderate | Low | Serious | Historical-control design; hypothesis-generating evidence for wearable-guided rehabilitation. |
| Yao et al., 2025 [13] | Moderate | Moderate | Low | Low | Low | Moderate | Low | Moderate | Prospective association evidence; no wearable-driven intervention. |
| Finet et al., 2023 [14] | Moderate | Moderate | Low | Low | Low | Moderate | Low | Moderate | Mobility-metric evidence; limited linkage to clinical outcomes. |
| Finley et al., 2020 [15] | Serious | Moderate | Low | Low | Low | Moderate | Low | Serious for causal inference | Single-arm feasibility evidence; not designed to estimate clinical effectiveness. |
| Finley et al., 2021 [16] | Serious | Moderate | Low | Low | Low | Low | Moderate | Serious for causal inference | Companion proof-of-concept report; not independent confirmatory evidence. |

Supplementary Table S2C. Descriptive appraisal for measurement-agreement study

| Study | Study type | Measurement reference | Data completeness / feasibility | Clinical-effect endpoint | Main limitation | Interpretation |
| --- | --- | --- | --- | --- | --- | --- |
| Wang et al., 2025 [17] | Development and usability / measurement-agreement study | Electronic health record vital signs | Adequate for measurement-feasibility interpretation | No comparative clinical-effect endpoint | No escalation algorithm tested; indirectness for clinical benefit | Measurement-feasibility evidence only; not assessed as an intervention-effect study. |

**Note:** RoB 2 was used for randomized trials, and ROBINS-I was used for nonrandomized comparative, observational, and single-arm studies. For single-arm feasibility studies, the judgment reflects risk of bias for causal inference rather than feasibility alone. Wang et al. was appraised descriptively because it did not estimate a comparative treatment effect. Finley et al., 2020 and Finley et al., 2021 were treated as companion reports from the same Dartmouth preoperative wearable-supported exercise research program and were not double-counted as independent evidence in the certainty assessment.

Supplementary Table S3. Detailed outcome extraction from included reports

| Study | Device/system and phase | Outcome domain | Key numerical results | Interpretation |
| --- | --- | --- | --- | --- |
| Patel et al., 2023 [11] | Fitbit-based Move For Surgery; preoperative | Prolonged length of stay; hospital stay; chest tube duration; feasibility | Prolonged LOS >5 days: 7% vs 24% (3/45 vs 12/50; *p=*0.021). Mean hospital stay: 2.67 vs 4.44 days; mean difference -1.77 days (95% CI, -2.90 to -0.65; *p*=0.002). Chest tube duration: 2.93 vs 5.46 days (*p*=0.076). Fitbit wear: 94.4% of trial time. | Randomized evidence for selected objective in-hospital endpoints; multicomponent intervention prevents isolation of wearable-specific effect. |
| Yang et al., 2025 [10] | Digital chest drainage + Amazfit; postoperative | Postoperative LOS; chest tube duration; sleep; ambulation; pulmonary complications | Postoperative LOS: 77.55±32.89 vs 107.02±124.87 h (*p*=0.012). Chest tube duration: 50.52±28.73 vs 73.80±115.90 h (*p*=0.028). PPCs: 7.8% vs 4.0% (*p*=0.157). | Randomized thoracic/pulmonary resection evidence; indirect for lung cancer-specific conclusions and not wearable-specific. |
| Lee et al., 2024 [12] | Fitbit Versa intervention; preoperative to 6 months | Daily steps; vigorous physical activity; 6MWD; HRQOL/symptoms | At 6 months: steps 12,321 vs 10,118 (*p*=0.007); vigorous physical activity 33.6 vs 18.5 min (*p*=0.003); no significant between-group difference in 6MWD. | Historical-control design; suggests improved free-living activity but causal certainty is limited. |
| Yao et al., 2025 [13] | Mi Band 5; preoperative and POD1-3 | Step count; LOS; SF-12 PCS; pain; complications | Preoperative steps vs LOHS: *r*=-0.146 (*p*=0.023). POD1 steps vs LOHS: *r*=-0.172 (*p*=0.018). POD1 steps vs 1-month PCS change: r=0.186 (*p*=0.013). Complications: 18/244 (7.4%); PPCs: 10/244 (4.1%). | Prospective observational association evidence; effect sizes modest and vulnerable to confounding/reverse causality. |
| Finet et al., 2023 [14] | ActiGraph GT3X; POD1-5 | Early postoperative mobility; activity intensity; sedentary behavior | 60 enrolled; 56 had at least one valid day. Daily steps and mean cadence did not significantly change during POD1-4; light-intensity activity increased; prolonged sedentary bouts decreased. Acceptability median score 10/10. | Supports multidimensional mobility assessment; step count alone may be insufficient for early recovery characterization. |
| Finley et al., 2020 [15] | Garmin Vivoactive HR; preoperative | Feasibility; synchronization; acceptability | 30 enrolled; 79% completed preoperative activities; 71% successfully synchronized the device; wearable data transmitted on 70% of preoperative days. | Single-arm feasibility evidence; no causal clinical-effect estimate. |
| Finley et al., 2021 [16] | Garmin Vivoactive HR; preoperative | MVPA; 6MWD; exercise adherence | 18 with device data. MVPA 20.4 min/day; 30 min/day target achieved on 16.4% of preoperative days. Mean 6MWD change +13.8 m (*p*=0.14); 8/17 (47%) achieved >=14 m improvement. | Companion proof-of-concept evidence; hypothesis-generating only. |
| Wang et al., 2025 [17] | HUAWEI WATCH D + ePRO; in-hospital | Physiologic agreement; outlier detection; ePRO integration | 288 NSCLC patients. Bias vs EHR: temperature 0.02°C; heart rate 0.26 bpm; SpO2 -0.06%. Wearable monitoring captured more measurements and outlier events than episodic ward recording. | Measurement-feasibility evidence; no comparative clinical-effect endpoint. |

**Abbreviations:** ePRO, electronic patient-reported outcome; EHR, electronic health record; HRQOL, health-related quality of life; LOHS, length of hospital stay; LOS, length of stay; MVPA, moderate-to-vigorous physical activity; PCS, physical component score; POD, postoperative day; PPCs, postoperative pulmonary complications; 6MWD, six-minute walk distance.

Supplementary Table S4. Outcome-level qualitative certainty assessment

| Outcome domain | Starting point | Risk of bias | Inconsistency | Indirectness | Imprecision | Final qualitative certainty | Explanation |
| --- | --- | --- | --- | --- | --- | --- | --- |
| Prolonged length of stay | RCT high | Downgraded | Not serious | Not serious | Some concern | Moderate | Single RCT with objective endpoint; downgraded for single-center design, early stopping/baseline imbalance concerns, and multicomponent intervention. |
| Postoperative hospital stay after digital drainage | RCT high | Downgraded | Not serious | Downgraded | Some concern | Low-to-moderate | Open-label RCT in mixed pulmonary resection population; indirect for lung cancer-specific and wearable-specific conclusions. |
| Step count and length of stay association | Observational low | Serious concern | Not serious | Not serious | Some concern | Low | Weak correlations and residual confounding/reverse causality remain plausible. |
| Chest tube duration | RCT high | Downgraded | Some concern | Downgraded | Some concern | Low-to-moderate | Randomized evidence available, but Yang et al. was mixed pulmonary resection and Patel et al. showed a nonsignificant trend. |
| Postoperative mobility and activity intensity | Mixed / observational low | Serious concern | Serious concern | Some concern | Some concern | Low | Directionally consistent signals but heterogeneous devices, metrics, time windows, and intervention contexts. |
| Sedentary behavior | Observational low | Some concern | Not serious | Not serious | Some concern | Low | Single observational accelerometry study with no control group and limited clinical outcome linkage. |
| Sleep recovery | RCT high | Downgraded | Not assessable | Downgraded | Some concern | Low | Secondary/exploratory sleep outcome within digital drainage intervention; not wearable-specific. |
| Functional capacity, 6MWD | Mixed / observational low | Serious concern | Some concern | Some concern | Some concern | Low | Small single-arm study and historical-control trial; inconsistent effect on clinic-based functional capacity. |
| HRQOL, dyspnea, and pain | Mixed / observational low | Serious concern | Some concern | Some concern | Some concern | Low | Patient-centered outcomes were secondary and variably defined; vulnerable to confounding and lack of blinding. |
| Physiologic agreement and continuous monitoring feasibility | Measurement study | Some concern | Not serious | Downgraded for clinical benefit | Some concern | Low for clinical benefit; moderate for measurement feasibility | Strong agreement signal, but no comparative clinical-effect endpoint or tested escalation algorithm. |
| Feasibility and implementation | Feasibility evidence | Some concern | Some concern | Some concern | Some concern | Low-to-moderate | Feasibility was generally consistent but based on selected participants, single-center settings, and variable platforms. |
| Complications and safety endpoints | Mixed evidence | Serious concern | Serious concern | Some concern | Serious concern | Very low | Sparse events, limited power, heterogeneous definitions, and complications were not consistently primary endpoints. |

**Note:** Certainty was qualitatively informed by GRADE principles rather than formal meta-analytic GRADE because quantitative pooling was not performed. Judgments considered study design, risk of bias, consistency, directness, precision, and whether each outcome represented clinical effectiveness, feasibility, or measurement agreement.

Abbreviations: GRADE, Grading of Recommendations Assessment, Development and Evaluation; HRQOL, health-related quality of life; RCT, randomized controlled trial; 6MWD, six-minute walk distance.
